# High-throughput platform for label-free sorting of 3D spheroids using deep learning

**DOI:** 10.3389/fbioe.2024.1432737

**Published:** 2024-12-09

**Authors:** Claudia Sampaio da Silva, Julia Alicia Boos, Jonas Goldowsky, Manon Blache, Noa Schmid, Tim Heinemann, Christoph Netsch, Francesca Luongo, Stéphanie Boder-Pasche, Gilles Weder, Alba Pueyo Moliner, Roos-Anne Samsom, Ary Marsee, Kerstin Schneeberger, Ali Mirsaidi, Bart Spee, Thomas Valentin, Andreas Hierlemann, Vincent Revol

**Affiliations:** ^1^ Automated Sample Handling Group, CSEM SA Centre Suisse d’Electronique et de Microtechnique, Neuchâtel, Switzerland; ^2^ Bio Engineering Laboratory, Department Biosystems Science and Engineering, ETH Zurich, Basel, Switzerland; ^3^ Department of Clinical Sciences, Faculty of Veterinary Medicine, Utrecht University, Utrecht, Netherlands; ^4^ Kugelmeiers Ltd., Erlenbach, Switzerland

**Keywords:** automation, high-throughput sorting, multi-cellular spheroids, 3D bioprinting, machine learning, transfer learning, tissue engineering

## Abstract

End-stage liver diseases have an increasing impact worldwide, exacerbated by the shortage of transplantable organs. Recognized as one of the promising solutions, tissue engineering aims at recreating functional tissues and organs *in vitro*. The integration of bioprinting technologies with biological 3D models, such as multi-cellular spheroids, has enabled the fabrication of tissue constructs that better mimic complex structures and *in vivo* functionality of organs. However, the lack of methods for large-scale production of homogeneous spheroids has hindered the upscaling of tissue fabrication. In this work, we introduce a fully automated platform, designed for high-throughput sorting of 3D spheroids based on label-free analysis of brightfield images. The compact platform is compatible with standard biosafety cabinets and includes a custom-made microscope and two fluidic systems that optimize single spheroid handling to enhance sorting speed. We use machine learning to classify spheroids based on their bioprinting compatibility. This approach enables complex morphological analysis, including assessing spheroid viability, without relying on invasive fluorescent labels. Furthermore, we demonstrate the efficacy of transfer learning for biological applications, for which acquiring large datasets remains challenging. Utilizing this platform, we efficiently sort mono-cellular and multi-cellular liver spheroids, the latter being used in bioprinting applications, and confirm that the sorting process preserves viability and functionality of the spheroids. By ensuring spheroid homogeneity, our sorting platform paves the way for standardized and scalable tissue fabrication, advancing regenerative medicine applications.

## 1 Introduction

End-stage liver failure is a life-threatening condition resulting in the complete loss of liver function, which led to more than 1.4 million deaths worldwide in 2019 (Liu and Chen, 2022). Currently, the only effective treatment is liver transplantation. Although the global number of liver transplants has been increasing in the last 3 decades (Terrault et al., 2023), the number of patients on waiting lists has increased three times faster (Kupiec-Weglinski, 2022) and is expected to keep growing due to population growth, aging, and increasing impact of alcohol-related liver disease (GBD, 2017 Cirrhosis Collaborators, 2020). Redefining donor criteria will not sufficiently increase the pool of liver donors, so that there is an urgent need to find alternative solutions to donor organs.

Regenerative medicine and tissue engineering are seen as promising solutions to revolutionize transplantation medicine and provide innovative approaches for regenerating or replacing severely diseased organs (Petrosyan et al., 2022). The primary challenge of *in vitro* tissue engineering lies in accurately reproducing the structural and cellular complexity of *in vivo* organs while ensuring their full functionality. Bioprinting technology addresses this challenge by enabling precise and reproducible spatial control over the deposition of cells and supporting material, thereby facilitating the reproduction of structural organ features on a large scale (Ramadan and Zourob, 2021). Concurrently, the development of multi-cellular spheroids, composed of multiple organ-specific cell types, enables to closely mimic the physiology and functionality of native tissue. The ability of bioprinting to fuse multi-cellular spheroids further holds promise for fabricating larger, functional tissues (Banerjee et al., 2022). Despite anticipating these advantages since 2009 (Mironov et al., 2009), the fabrication of large-scale organs using bioprinting of spheroids remains hindered by a persistent challenge: the lack of production methods to obtain large quantities of homogeneous spheroids (Banerjee et al., 2022).

Automation and parallelization of spheroid production significantly improved spheroid reproducibility and production speed. Various culturing platforms have been developed, leveraging on microfluidic devices (Schuster et al., 2020), lab automation systems (Brandenberg et al., 2020; Renner et al., 2020), and standardized culture plates (Gri3D, Sun bioscience; Akura^TM^ plates, InSphero; Sphericalplate 5D, Kugelmeiers). While these platforms offer increased throughput at a cost-effective rate, the produced tissue spheroid populations exhibit a persistent degree of inhomogeneity, which limits their direct use for bioprinting applications.

Sorting platforms offer a promising opportunity to enhance the homogeneity of a spheroid population and improve transferability compared to traditional production platforms. These platforms enable automated imaging, analysis, and sorting of spheroids, guided by user-defined sorting criteria. Most commercially available systems use a pick-and-place approach to sort individual spheroids based on morphological parameters such as size and shape extracted from microscopic images (Cell Handler, Yamaha; CellCelector, Sartorius; CellPicker. Shimadu). To enhance sorting throughput, alternative systems use faster handling and analysis methods, such as flow cytometers and laser-based fluorescence readouts (COPAS FP, Union Biometrica). However, these systems are primarily tailored for drug screening applications and, consequently, do not meet the requirements for bioprinting organ transplants, which necessitates high quantities of label-free spheroids. Current systems are thus either too slow, due to non-optimized platform movements for individual spheroid handling, or depend on invasive analysis methods, such as fluorescent labels.

Moreover, bioprinting applications rely on highly specific and diverse sorting criteria based on distinct morphological characteristics and spheroid viability. These requirements pose challenges for conventional sorting systems that are limited to simple morphological parameters such as size as a sorting criterion or assess spheroid viability using fluorescent labels. In recent years, deep learning has proven to be a potent tool to enhance image analysis, leveraging its capacity to precisely identify unique patterns within large image datasets (Ching et al., 2018; Renner et al., 2021). In particular, deep learning models have demonstrated their efficacy in enhancing spheroid segmentation (Gritti et al., 2021), detection (Lacalle et al., 2021), tracking (Matthews et al., 2021), and characterization (Bian et al., 2021). Despite the adoption of deep learning models for spheroid segmentation in sorting systems (Grexa et al., 2021; Du et al., 2023), this approach remains constrained to standard morphological features such as size and shape and requires large datasets which are not always easy to produce for complex biological tissues. However, the use of deep learning for direct spheroid classification is a promising tool to extract complex patterns from images, increasing reliability for bioprinting applications.

Addressing these limitations, the European project OrganTrans was aimed at developing a standardized process for fabricating implantable liver constructs using bioprinting of tri-cellular liver spheroids, made of three different cell types. The collaborative project OrganTrans included seven studies for the establishment of all fabrication steps, including the development of a multi-cellular model for liver transplantation, an optimized bioink for spheroid network formation, the automated sorting of spheroids and a bioprinting process. The scope of this study was limited to developing an automated spheroid sorting solution for over 12,500 tri-cellular liver spheroids to print a single 0.5 cm^3^ liver construct. For this purpose, an automated, high-throughput sorting system was essential to select homogeneous, high-quality spheroids suitable for bioprinting.

Within the framework of OrganTrans, we developed a fully automated sorting platform for high-throughput sorting of liver spheroids. This sorting platform seamlessly integrates image acquisition, image analysis, and transfer of individual spheroids directly from the culture plate into a single, streamlined process. We propose an alternative sorting workflow to that of standard pick-and-place platforms, which provides enhanced sorting speed while enabling precise handling of individual spheroids and acquisition of high-quality images. The compact platform design ensures compatibility with standard safety cabinets and culture plates, allowing for adaptability to various sorting processes. Moreover, we successfully trained a deep learning model for spheroid detection and classification, enabling label-free characterization of spheroids for bioprinting applications. Additionally, we show that transfer learning proves effective in training deep learning models even when only a small dataset of spheroid images is available. We demonstrate the high-throughput sorting process for two types of liver spheroids, mono-cellular HepG2 spheroids and tri-cellular spheroids, the latter being utilized in the OrganTrans project. The developed sorting platform facilitates the selection of suitable spheroids when large quantities of standardized 3D tissues are needed, ultimately enabling their use in a clinical setting for regenerative medicine applications.

## 2 Materials and methods

### 2.1 Components of the SpheroidSorter platform

The SpheroidSorter platform included two distinct fluidic systems: a picking system for individual removal of low-quality spheroids, and a harvesting system for bulk collection of high-quality spheroids that remained in the plate after picking. The picking system consisted of a low-pressure gear pump (mzr-4622, HNP Mikrosysteme, Schwerin, Germany) connected via silicon tubing (ID 1.0 mm, OD 2.0 mm, Carl Roth, Karlsruhe, Germany) to a 1.5 mL Eppendorf tube (BGB Analytik, Adliswil, Switzerland), in which low-quality spheroids were collected. To prevent inadvertent entry of spheroids and cells into the pump, we placed a sterile filter of 0.22 µm pore size (Guangzhou Jet Bio-Filtration, Guangzhou, China) between the pump and the Eppendorf tube. A metal tube cap (P-CAP series, Fluigent, Le Kremlin-Bicêtre, France) ensured air-tight sealing of the Eppendorf tube, and its connection via an Ethylene tetrafluoroethylene (ETFE) tubing connector (P-663, Idex Corporation, Lake Forest, United States) to a capillary tube (250 μm ID, 360 μm OD, TSP Standard FS tubing, BGB Analytik, Adliswil, Switzerland) that was used to pick up low-quality spheroids. The picking end of the capillary tube was inserted into a sterile needle (G19x1 1/4″, B.Braun, Melsungen, Germany) to prevent it from breaking, and the assembly was referred to as “picking needle.” The size of the capillary tube was chosen to enable high-precision picking of 150 μm-diameter spheroids. To enable precise vertical movement of the picking needle, we used an automated linear stage (VT-80, Physik Instrumente, Karlsruhe, Germany). A customized needle holder was designed to ensure stable attachment of the picking needle to the linear stage using two square magnets (Q-10-10-04-K, Webcraft, Uster, Switzerland). The linear stage ensured precise vertical movement of the needles for both picking and harvesting processes. The harvesting system included two pumps: a peristaltic pump (KPP_DA-S10, Kamoer Fluid Tech, Shanghai, China) and a vacuum pump (Tops Industry & Technology, Changsha, China). The peristaltic pump dispensed culture media through a PTFE tubing (ID 2.0 mm, OD 4.0 mm, Maagtechnic, Dübendorf, Switzerland) and a 0.5 mm large needle (23ga × 1.0″ Sky Blue Blunt Tip Dispensing Fill Needles, CML Supply, Lexington, United States) in the culture well during harvesting, which facilitated the dislodging of spheroids. The vacuum pump simultaneously harvested high-quality spheroids in bulk from the plate through a needle of 1.6 mm inner diameter (501,137, Vieweg Dosier und Mischtechnik, Kranzberg, Germany). A 3D-printed custom-made needle holder was designed to enable the positioning of the media-dispensing needle parallel to the spheroid-collecting needle. The fluidic system for harvesting included silicon tubing (ID 2.0 mm, OD 4.0 mm), polytetrafluoroethylene (PTFE) tubing (ID 2.0 mm, OD 3.0 mm, Carl Roth, Karlsruhe, Germany) and male Luer Lock connectors (BGB Analytik, Boeckten, Switzerland). The spheroids were collected into two 50 mL Falcon tubes (Greiner Bio-One, Kremsmünster, Austria), each equipped with 3D-printed custom-made lids. Waste was collected into a 250 mL glass bottle with a 3D-printed custom-made cap, including an O-ring (Kubo, Illnau-Effretikon, Switzerland) to ensure airtightness. The SpheroidSorter was designed so that only the culture plate would be moving horizontally using a motorized XY-stage (M-687.UN XY Stage, Physik Instrumente (PI) GmbH & Co. KG, Karlsruhe, Germany) to prevent misalignment between the picking needle and the optical setup during sorting. Finally, the main structure of the SpheroidSorter platform was made using standard aluminum construction profiles and connectors from Item Industrietechnik (Solingen, Germany).

### 2.2 3D printing

The custom-made structural components of the SpheroidSorter platform were fabricated using an Original Prusa i3 MK3S printer (Prusa Research, Prague, Czech Republic), based on fused deposition modelling (FDM) technology. We used Prusament PLA (Polylactic Acid) filaments and a 0.15 mm layer height for all prints to ensure precision and reliability. The two Falcon tube lids and the glass bottle cap were fabricated with a Preform Form 3D printer (Formlabs, Somerville, United States). This printer operates on stereolithography (SLA) technology, which allowed us to fabricate elaborate components while ensuring airtightness, essential for our vacuum-based system. Specifically, the glass bottle cap was printed using Rigid 4000 resin (Formlabs, Somerville, United States), while the Falcon tube lids were crafted with BioMed Clear resin (Formlabs, Somerville, United States) to ensure biocompatibility. All our computer-aided design (CAD) models were developed using Solidworks software.

### 2.3 Software

The entire sorting process was controlled by a proprietary software platform designed to integrate and control multiple hardware components via a centralized graphical user interface (GUI). The software was written in C++ and enabled the integration of all hardware components including a camera, a motorized XY-stage, a motorized Z-stage, a gear pump, a peristaltic pump, a vacuum pump, and a deep-learning module. Our software was tailored for two types of culture plates: the Sphericalplate 5D (SP5D) 24-well plate and 6-well plate (Kugelmeiers Ltd., Erlenbach, Switzerland). Within the GUI, various critical parameters could be adjusted, such as the flow rates for picking and harvesting processes, and the needle height for picking. Two additional workflows were implemented enabling calibration and cleaning of the platform. The calibration process was customized for each specific culture plate and consisted of two steps: 1. vertical alignment between picking needle and brightfield microscope, and 2. vertical alignment between harvesting needle and culture plate. The first step was needed for precise removal of individual spheroids through real-time imaging, whereas the second step enabled optimization of the harvesting path with respect to well size, increasing harvesting efficiency. The cleaning process was developed for rinsing of the entire harvesting fluidic system after sorting. Our software provided precise control over each sorting step, allowing for independent or collective execution of the sorting workflow over the entire plate or focusing on a specific well.

### 2.4 Cell culture

The human hepatocellular carcinoma (HepG2, HB-8065™, ATCC) cells were maintained in HepG2 medium containing low glucose Dulbecco’s Modified Eagle Medium (DMEM) supplemented with 10% (v/v) fetal bovine serum (FBS, S0615, Bioswisstec) and 1% (v/v) penicillin-streptomycin (P4333, Sigma-Aldrich, Zug, Switzerland). Cells were passaged at 80% confluency following routine protocols. Briefly, the cells were trypsinised, centrifuged and counted using trypan blue solution 4% (w/v) (Sigma-Aldrich, Zug, Switzerland) and split into 25 cm^2^ culture flasks (Guangzhou Jet Bio-Filtration, Guangzhou, China).

### 2.5 Spheroid culture

HepG2 spheroids were formed and cultured in HepG2 medium in SP5D 24-well plates by seeding 200 cells per micro-well (i.e., 150,000 cells per well, with each well containing 750 micro-wells). The medium was changed every 2-3 days. After 5 days in culture, the HepG2 spheroids were harvested for experimentation.

The tri-cellular liver spheroids were provided by Dr. Bart Spee, Utrecht University, the Netherlands. Tri-cellular spheroids were composed of human umbilical cord endothelial cells (HUVECs), mesenchymal stem cells (MSCs) and hepatic cells. Tri-cellular spheroids were cultured in SP5D 24-well plates in tri-cellular spheroid media (based on Advanced DMEM/F12, EGM-2 containing B27) and harvested on day 5 for experimentation.

### 2.6 Image acquisition

All training images for the neural network were acquired using the optical setup of the SpheroidSorter with no subsequent post-processing. The optical setup consisted of a custom-made brightfield microscope with a uEye IDS camera (UI-3080CP-M-GL Rev.2, IDS Imaging, Obersulm, Germany), connected to a 50 mm long C-mount extension tube (Thorlabs, Newton, United States), an Optotune lens (EL-10-30-C(i)-VIS-LD, Optotune, Dietikon, Switzerland), a Female M22 × 0.75 to Male C-Mount Adapter (34-770, EO Edmund Optics, Barrington, United States), and a 25 mm, f/4 Ci Series Fixed Focal Length Lens (85-358, EO Edmund Optics, Barrington, United States). Two orthogonally placed Manual 1” (25 mm) Linear Translation Stages (Thorlabs, Newton, United States) enabled manual alignment of the microscope with the picking needle. Optical rails (50 mm) from Thorlabs were used to hold the custom-made microscope in place. A diffuse ring light FLKR-Si100 (Falcon Illumination, Untereisesheim, Germany) was positioned above the XY-stage for homogeneous lighting conditions. A 3-D printed custom-made part attached the ring light and the linear stage to the platform structure.

### 2.7 Network structure of the YOLOv5

The YOLOv5 architecture consists of three main parts: (i) the backbone for feature extraction from the input image, (ii) the neck that fuses features from different levels of the backbone to generate feature maps for the detection of objects of different size, and (iii) the head, which generates the final output, consisting of a multi-dimensional array that includes object class, class confidence, box coordinates, and corresponding width and height (Ultralytics, 2024). For this study we used the default YOLOv5 release v6.0 structure (Liu et al., 2022) and the small model size YOLOv5s. The YOLOv5 backbone first introduces the structure called CSP-Darknet53 (Wang et al., 2019). This Cross Stage Partial (CSP) network is designed to extract deep features from the input data which reduces computation compared to YOLOv3. The release v6.0 further optimized the neck of the YOLOv5 model by integrating the Spatial Pyramid Pooling Fusion (SPPF) module and the Path Aggregation Network (PANet) structure. The SPPF module is a pooling layer that enhances the receptive field of the model by applying max-pooling layers of sizes 1 × 1, 5 × 5, 9 × 9, and 13 × 13 and eliminates the fixed-size constraint of the network. The PANet structure improves object detection and class recognition by preserving spatial information and employs a feature pyramid network with multiple top-down and bottom-up layers (Liu et al., 2018). The head of the model consists of fully connected networks of sizes 19 × 19, 38 × 38, and 76 × 76 (Redmon et al., 2018), which integrate and interact with feature maps of different scales. These networks have been improved in the YOLOv5 v6.0 to enhance the detection of objects of varying sizes and the efficiency of multi-scale feature handling.

### 2.8 Dataset generation

We created two datasets for the training of the detection and classification network of the SpheroidSorter platform. All images (2,054 × 2,456 pixels, 24 bits depth) were generated by scanning SP5D culture plates, containing either HepG2 or tri-cellular spheroids, using the imaging system of the SpheroidSorter platform.

The first dataset consisted of 516 images of tri-cellular spheroids imaged after 5 days of culture. Spheroids were labeled and classified by experts, assigning a “high-quality” or “low-quality” label based on spheroid size, shape, and morphological regularity/homogeneity. The tri-cellular dataset consisted of 4,176 spheroids labeled as “high-quality” and 194 deemed “low-quality.”

The second dataset consisted of 1,395 images of HepG2 spheroids imaged after 5 days of culture. A portion of these spheroids was treated with 2.5% (v/v) DMSO during culture to obtain an artificially induced low-quality dataset. This deliberate intervention facilitated the creation of a more balanced dataset, crucial for improved classification accuracy. To facilitate the labeling of this large HepG2 dataset, we trained a YOLOv5 network using manually labeled images with bounding boxes around spheroids for precise localization. This localization model was integrated into our labeling tool, enabling automatic spheroid detection. For spheroids exposed to DMSO, a “low-quality” label was automatically assigned, whereas non-DMSO exposed spheroids were assigned a “high-quality” label. This labeling strategy enabled the fast generation of a large HepG2 dataset without the need of bioprinting experts. The obtained HepG2 dataset consisted of 12,599 “high-quality” spheroids and 6,530 “low-quality” spheroids.

### 2.9 Network training

For robust and accurate object detection and classification, we used the open-source YOLOv5 network. We integrated transfer learning in our training pipeline to improve classification accuracy of tri-cellular spheroids challenged by the small size of the dataset. Initially, we pre-trained the YOLOv5 network on a large HepG2 dataset, adhering to default parameters of YOLOv5, which also included data augmentation methods, as outlined in the literature (Jocher et al., 2022). The HepG2 dataset was partitioned into three sets: 70% for training (13,381 labels of which 66% were high-quality spheroids), 15% for testing (2,603 labels of which 64% were high-quality spheroids) and 15% for validation (3,145 labels of which 66% were high-quality spheroids). Subsequently, we fine-tuned the pre-trained model with a smaller tri-cellular dataset. During this second training phase, we strategically froze the initial ten layers of the network and adopted a weighted image selection approach. Notably, we tailored our data augmentation strategy by removing Blur, MedianBlur, ToGray and ImageCompression methods, while adjusting the p-value for Clahe, RandomBrightnessContrast, RandomGamma, ColorJitter and RGB Shift to 0.2. The tri-cellular dataset was partitioned into three sets: 80% for training (3,470 labels of which 95% were high-quality spheroids), 10% for testing (459 labels of which 97% were high-quality spheroids) and 10% for validation (441 labels of which 95% were high-quality spheroids).

### 2.10 Viability assays

Fluorescent Staining: To test the viability of spheroids throughout the sorting procedure, live cells in HepG2 spheroids were stained with green Fluorescein Diacetate (FdA, F7378, Sigma-Aldrich, Zug, Switzerland) and dead cells with Propidium Iodide (PI, P4170, Sigma-Aldrich, Zug, Switzerland). After manual and automated harvesting, HepG2 spheroids were transferred to 24-well plates in HepG2 medium and kept in an incubator (37°C, 5% CO_2_). For the staining process, spheroids were washed with phosphate buffered saline (PBS), pH 7.4 (ThermoFisher Scientific, Waltham, United States), mixed with 500 µL of staining solution (144 μM of FdA and 35.9 μM of PI in PBS) and incubated for 15 min (37°C, 5% CO_2_) in the dark. After incubation, spheroids were washed with PBS three times. A Spark Cyto plate reader (Tecan, Männedorf, Switzerland) was used to acquire brightfield images and detect fluorescence using FITC and Cy3 filters for FdA and PI respectively.

Tri-cellular spheroids were stained with LIVE/DEAD™ viability/Cytotoxicity Kit for mammalian cells (Invitrogen, Carlsbad, United States) to stain living cells with green fluorescent Calcein-AM (Ca/AM), and dead cells with red fluorescent ethidium homodimer-1 (EtBr). After manual and automated harvest, tri-cellular spheroids were embedded in 50 µL droplets of Matrigel Growth Factor Reduced (GFR) Basement Membrane (356,231, Corning, NY, United States) in 24-well plates with 1 mL of tri-cellular media. For the staining process, we removed any excess media from each well and added 400 µL of staining solution (4 µM of ETBr and 2 µM of Ca/AM in PBS). After 20 min of incubation at 37°C, the staining solution was removed and the resulting fluorescence signal was detected using EVOS™ Digital Color Fluorescence Microscope (Invitrogen, Carlsbad, United States).

ATP Assay: Adenosine triphosphate (ATP) levels were assessed using the CellTiter-Glo Luminescent Cell Viability Assay (Promega, Dübendorf, Switzerland) following the manufacturer’s protocol. After sorting, spheroids were seeded in an ultra-low attachment 24-well plates, and luminescence measurements were normalized based on the spheroid count in each well. The luminescence signal was detected with a luminescence plate reader (Tri-Star2, LB 942, Berthold Industries, Bad Wildbad, Germany).

LDH Assay: Lactate dehydrogenase (LDH) release was quantified using the CytoTox 96^®^ Non-Radioactive Cytotoxicity Assay Technical Bulletin (Promega, Dübendorf, Switzerland). We harvested 50 μL cell-free supernatant from a single well of ultra-low attachment 24-well plates, in which spheroids had been seeded post-sorting and mixed with 50 µL of CytoTox 96^®^ Reagent in another well of a 96-well plate. The plate was incubated at room temperature for 30 min while being protected from light. We stopped the reaction by adding 50 µL of stop solution. We measured the absorbance at a wavelength of 490 nm using a standard plate reader (CLARIOstar Plus, BMG Labtech, Ortenberg, Germany).

### 2.11 Image analysis

Microscopy images were analyzed with ImageJ. Area and circularity were calculated by using ImageJ integrated tools after manual spheroid segmentation.

### 2.12 Statistical analysis

Statistical analysis was performed using GraphPad Prism. Differences between experimental groups were assessed using a two-tailed unpaired t-test with Welch’s correction (*, p < 0.05; **, p < 0.005; ***, p < 0.0005). All results are shown as mean values ±standard error of mean (SEM).

## 3 Results

### 3.1 Workflow of the SpheroidSorter platform

**FIGURE 1:**
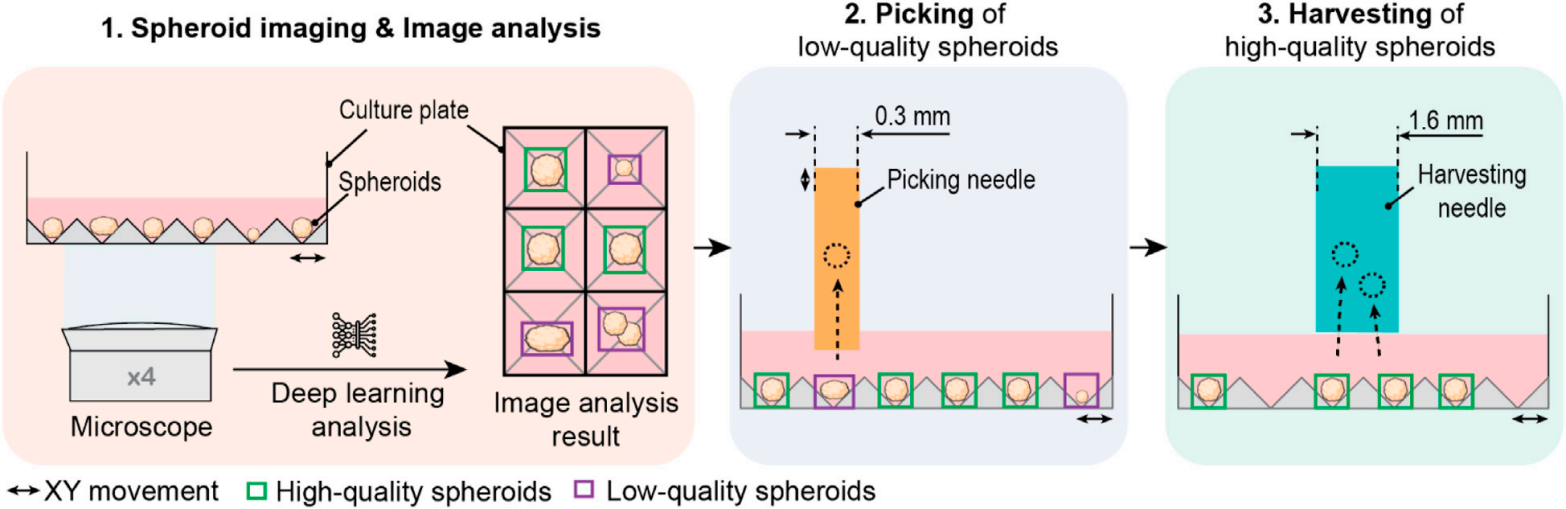
Schematic of a 3-steps workflow of the SpheroidSorter. The workflow of the SpheroidSorter is divided into three steps: 1. Spheroid imaging and image analysis. An inverted bright-field microscope with ×4 magnification images spheroids in the culture plate. In parallel, each spheroid is detected and analyzed by a deep learning algorithm and classified with a “low-quality” label (purple) or “high-quality” label (green). 2. Individual picking of low-quality spheroids. Spheroids labelled as low-quality are individually removed by a picking needle of 0.3 mm diameter. 3. Harvesting of high-quality spheroids. High-quality spheroids are harvested in bulk by a harvesting needle of 1.6 mm diameter.

We developed a fully automated spheroid sorter that integrated the entire sorting process of image acquisition, image analysis, and physical sorting in a single platform. We devised the workflow of the “SpheroidSorter” platform as three consecutive work steps, schematically described in Figure 1. In the first step, automated spheroid imaging and simultaneous image analysis were performed over the entire spheroid culture plate, containing 8,000 spheroids (SP5D 24-well plate). Spheroids were imaged using an inverted bright-field microscope with ×4 magnification. We designed the inverted microscope to have an extended field of view of 1.8 × 2.2 mm, enabling simultaneous imaging of twelve spheroids, significantly reducing imaging time of the entire culture plate. In parallel, image analysis was performed by a deep learning algorithm, which detected, analyzed, and classified spheroids as “low-quality” (purple box, Figure 1) and “high-quality” (green box) tissues. The algorithm was trained with images of spheroids labeled by experts according to required characteristics for bioprinting applications. In step two, low-quality spheroids from the culture plate were selectively removed with a 0.3 mm-diameter capillary picking needle, as shown in Figure 1. We chose a needle diameter of 0.3 mm to match spheroid size and allow for accurate and efficient picking of individual low-quality spheroids without disturbing high-quality spheroids in close vicinity. In step three, all remaining high-quality spheroids were collected in bulk with a harvesting needle of 1.6 mm diameter.

The workflow of the SpheroidSorter platform was controlled using Visard (CSEM, 2024) a proprietary software platform developed by CSEM and designed to control multiple hardware components from a centralized graphical user interface (GUI). The centralized GUI enabled independent control of each step of the SpheroidSorter workflow and live visualization of image analysis results.

Simultaneous imaging of multiple spheroids, parallelization of image analysis, and bulk harvesting resulted in a substantial reduction in overall sorting time, directly correlated to the number of low-quality spheroids in the culture plate. Additionally, the integration of all three spheroid sorting steps into a single platform eliminated the need for manual handling throughout the entire process. The comprehensive integration rendered the system particularly well-suited for high-throughput screenings.

### 3.2 Design of the SpheroidSorter platform

**FIGURE 2:**
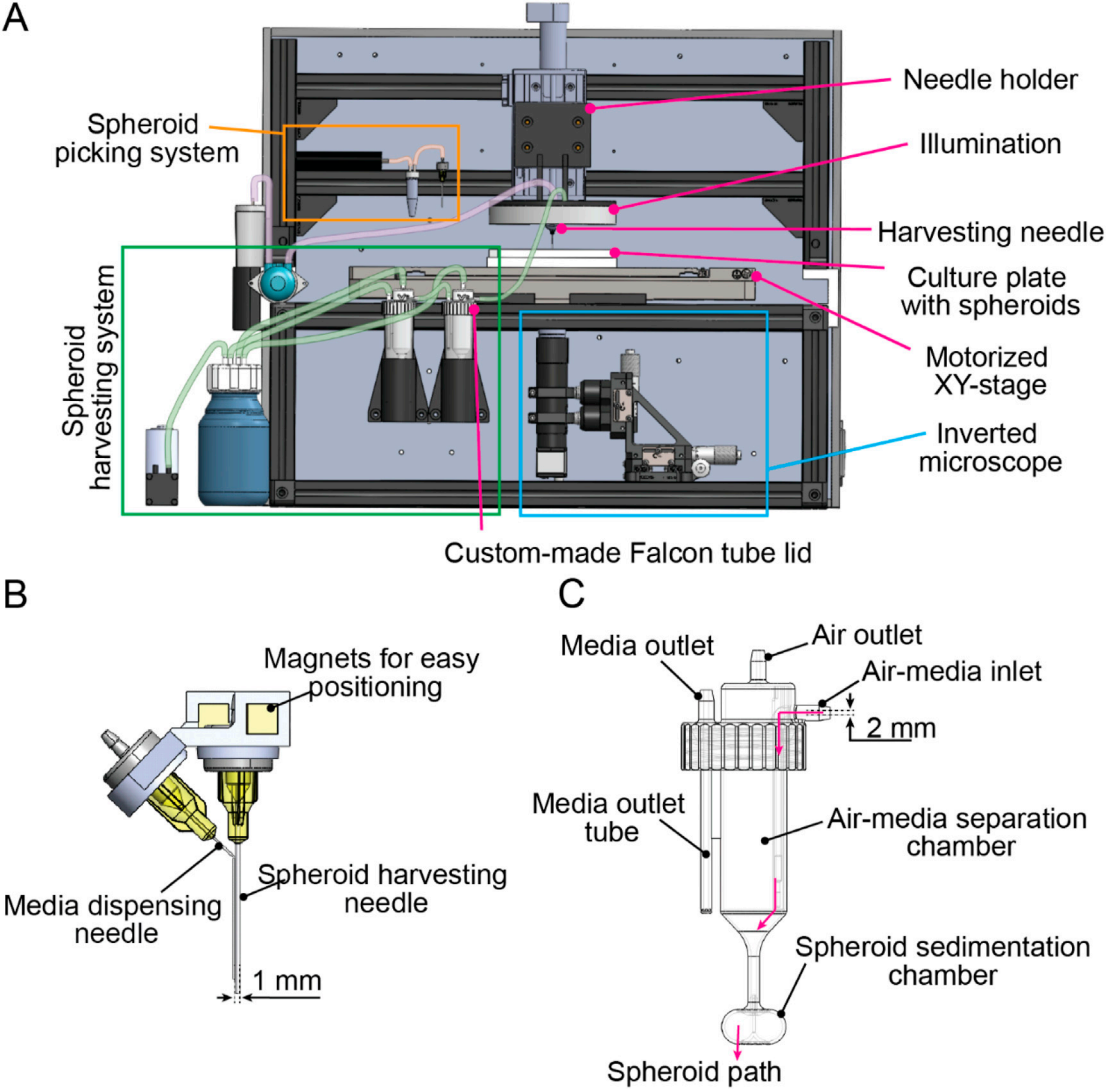
SpheroidSorter platform. **(A)** Schematic of the SpheroidSorter platform. The platform is organized around a moving XY-stage, containing the SP5D culture plate with spheroids. It includes an inverted microscope, a fluidic system for spheroid picking and a fluidic system for spheroid harvesting. **(B)** Harvesting needle. The harvesting needle consists of a pair of needles: a needle of 0.5 mm diameter for media dispensing and a needle of 1.6 mm diameter for spheroid harvesting. **(C)** Custom-made lid for Falcon tubes. High-quality spheroids are collected in a 3D-printed customized lid, promoting spheroid sedimentation and air-liquid separation. First, an “air-media separation chamber” eases air separation from media entering through the air-media inlet. The “spheroid sedimentation chamber” facilitates spheroid sedimentation at the bottom of the Falcon tube, hence preventing removal of high-quality spheroids when excess media is removed through the media outlet tube.

We designed the SpheroidSorter as a fully integrated and compact platform, that fits within standard laminar flow cabinets. As illustrated in Figure 2A, the compact platform design included an inverted microscope, a fluidic system for spheroid picking and a second fluidic system for spheroid harvesting. All three systems were arranged around a motorized XY-stage with 0.1 µm sensor resolution and ±0.3 µm bidirectional repeatability, enabling precise and repeatable positioning of the culture plate. The culture plate constituted as the only moving element in the XY plane. By minimizing movements of the picking needle, we successfully maintained vertical alignment between the needle and the microscope. Stable alignment between the two components ensured high precision during individual picking of low-quality spheroids supported by real-time imaging.

The picking system was positioned at the back of the platform and included a gear pump for high precision liquid handling with 2 μL minimal volume, and a 1.5 mL Eppendorf tube, in which low-quality spheroids were collected. During the picking process, the picking needle was fixed on a needle holder, connected to a motorized vertical Z-stage that enabled raising and lowering of the needle with 0.5 µm minimum incremental motion. The same needle holder was used during picking and harvesting phases in our workflow.

The harvesting needle consisted of a two-needle setup, schematically shown in Figure 2B. A small needle of 0.5 mm diameter was used to dispense culture media at a high flow rate into the microwells. Dispensing culture media during the harvesting facilitated the dislodgement of remaining high-quality spheroids from the microwells, increasing harvest efficiency and preventing drying of the well during harvest. Simultaneously, a larger needle of 1.6 mm diameter was used to harvest high-quality spheroids by aspirating the dislodged spheroids. We chose a 1.6 mm needle diameter to enable the simultaneous collection of multiple spheroids, and to minimize shear stress on spheroids.

The fluidic system for spheroid harvesting was equipped with a vacuum pump to induce negative pressure and enable collection of high-quality spheroids into two 50 mL Falcon tubes. To remove excess media from the two Falcon tubes without losing spheroids, we developed a customized 3D-printed lid for Falcon tubes. The customized lid, schematically shown in Figure 2C, was designed to facilitate both spheroid sedimentation and air-liquid separation by using centrifugal forces. We designed the customized Falcon lid to have a cylindrical chamber with a conical base, the “air-media separation chamber,” which created a first rotational flow. Harvested spheroids were aspirated through the inlet into the “air-media separation chamber,” where air was effectively separated from the harvested media. An air outlet located at the top of the air-media separation chamber enabled air to exit the Falcon tube using negative pressure from the vacuum pump. Subsequently, spheroids were aspirated into the “spheroid sedimentation chamber,” an ellipsoidal chamber where a second rotational flow promoted spheroid sedimentation at the bottom of the Falcon tube. By promoting spheroid sedimentation, we effectively reduced the risk of losing spheroids during the removal of excess media through the media outlet. The volume of removed media can be regulated by adjusting the length of the media outlet tube, allowing for the adjustment of the final spheroid concentration in the Falcon tube. Additionally, we connected two Falcon tubes in series to further reduce the risk of losing spheroids through the media outlet, thereby increasing harvesting efficiency.

Overall, we optimized the design of the platform to increase sorting accuracy and harvesting efficiency while keeping spheroids viable and providing a user-friendly platform for spheroid sorting.

### 3.3 Hardware performance

We evaluated the picking and harvesting efficiency of the SpheroidSorter platform using HepG2 spheroids, a well-characterized mono-cellular aggregate of hepatic cells. Chosen for their resemblance in size and organ type to the tri-cellular liver spheroids used in OrganTrans’ bioprinting application, HepG2 spheroids served as a representative benchmark. To assess the picking efficiency, we randomly selected 50 spheroids from three wells of two SP5D 24-well plates, each containing HepG2 spheroids cultured for 5 days. Picking needle calibration was performed once for each plate before picking. The system successfully picked the targeted spheroid with 91% efficiency, as shown in Table 1, taking 5 s per spheroid. We identified a few errors, including simultaneous picking of two spheroids (observed in 62% of cases) and failure to pick any spheroid.

**TABLE 1:** Time and efficiency of each process step of the SpheroidSorter platform. Process time and efficiency were evaluated using mono-cellular HepG2 spheroids cultured in SP5D 24-well plate (∼8,000 spheroids/plate). Time for spheroid imaging and harvesting was assessed for the complete culture plate. Picking time and efficiency were evaluated from the picking of n = 50 spheroids from 3 wells of 3 culture plates. Harvesting efficiency was evaluated by assessing the number of successfully recovered spheroids after the sorting process, n = 3 culture plates. Mean ± SEM.

Process step	Step time	Step efficiency
Picking of low-quality spheroids	5 s/spheroid	91% ± 6%
Harvesting of high-quality spheroids	20 min	92% ± 5%
Spheroid imaging and image analysis	30 min	NA

To assess the harvesting efficiency, three entire culture plates were automatically harvested using the SpheroidSorter. Across all three plates, an average of 92% of spheroids were successfully harvested within 20 min (Table 1). Of the unharvested spheroids, only 35% remained in the culture plate, while the remaining was found in the waste bottle.

To evaluate the SpheroidSorter’s performance against bioprinting requirements, including sorting speed and efficiency, partners established a minimum throughput of 1,500 spheroids sorted per hour. Notably, the SpheroidSorter platform efficiently sorted a culture plate of 8,000 spheroids, with 2% low-quality spheroids, in less than 2 h with a picking and harvesting efficiency exceeding 90%. Spheroid imaging and analysis accounted for 30 min (Table 1) of the complete sorting process. Overall, the platform enabled accurate and precise sorting of large quantities of spheroids, thereby rendering access to homogeneous spheroid populations.

### 3.4 Automated classification of liver spheroids using deep learning

#### 3.4.1 Training of the Yolov5 network

Sorting over 1,500 spheroids per hour for bioprinting applications requires rapid and automated analysis of large volumes of image data and multiple parameters analysis. We selected the YOLOv5 network (Jocher et al., 2022), a machine learning model that performs both object detection and classification on input images. This convolutional neural network outputs object class, class probabilities, and bounding boxes around detected objects. YOLOv5 is a one-stage object detection network with a light structure optimized for computation efficiency, which makes it an ideal tool for real-time analysis (Redmon et al., 2015). Moreover, studies have shown the superior accuracy of YOLOv5 compared to other fast object detection models, such as Faster-RCNN, SDD and Detectron-2, especially when using data augmentation technics (Wang et al., 2022; Kalbhor et al., 2023). Overall, its ease of training and implementation, made it an optimal solution for our automated sorting system.

**FIGURE 3:**
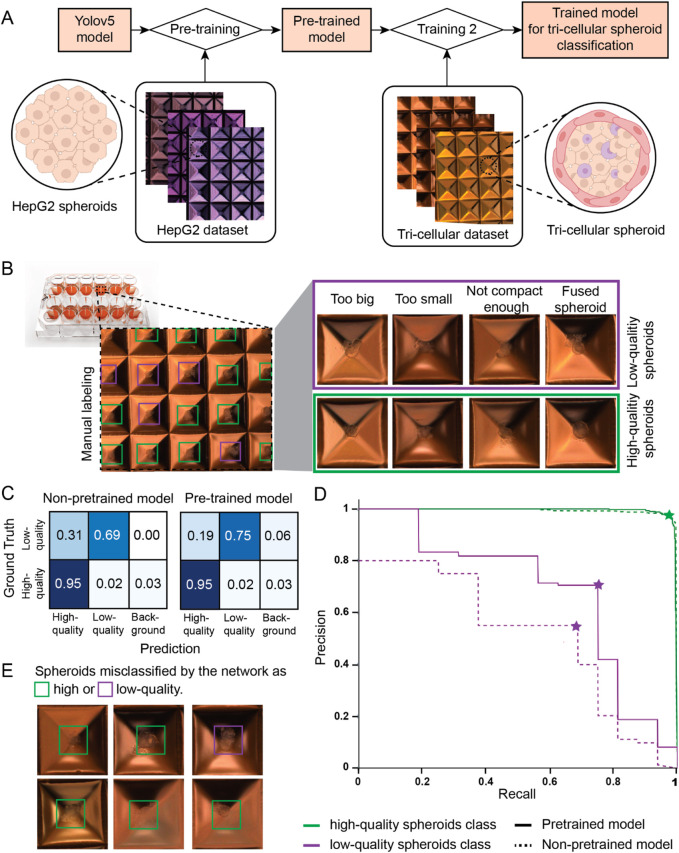
Training and classification of a deep learning model. **(A)** Schematic view of the designed training pipeline. A YOLOv5 model is pre-trained using a large image dataset of mono-cellular HepG2 spheroids. Subsequently, the pre-trained model is trained again on a smaller image dataset of tri-cellular spheroids, outputting the final model used for tri-cellular spheroid classification. **(B)** Schematic representation of the used manual labeling method. Images of high-quality (green box) and low-quality (purple box) spheroids based on size, shape, viability, compactness or aggregation are highlighted. **(C)** Confusion matrix for the two models obtained with different training pipelines: the YOLOv5 model was solely trained on the tri-culture dataset (non-pre-trained model) and the YOLOv5 model was trained using transfer learning (pre-trained model). **(D)** Precision-Recall curve for the high-quality (green) and low-quality (purple) classes evaluated for a model pre-trained with our training pipeline (solid line) and for a non-pre-trained model where only the tri-cellular dataset was used (dashed line). **(E)** Images of spheroids misclassified as high-quality (green box) and low-quality (purple box) by the pre-trained model.

Considering the limited availability of tri-cellular liver spheroids used for bioprinting, we incorporated transfer learning into our training pipeline, as described in Figure 3A. This approach facilitates learning tasks when only a small dataset is available. Transfer learning can be divided in two steps: first, a model is pre-trained on a large dataset for a similar classification task, then the obtained pre-trained model is fine tuned for the final classification task by training it a second time on the smaller dataset of interest. For our application, we decided to pre-train our model on a large dataset of HepG2 spheroids images. HepG2 spheroids were chosen for their ease of production in high-throughput and for their resemblance in shape and size to tri-cellular spheroids. Subsequently, we fine-tuned the resulting model by training it on a smaller dataset of tri-cellular spheroids, manually labeled by experts. To account for the small and unbalanced tri-culture dataset, we optimized the training by applying data augmentation methods, such as RGB shift and random Gamma selection, and selectively froze the initial ten layers during training (see Materials and methods section).

#### 3.4.2 Dataset generation

We created two custom datasets for the classification of spheroids according to bioprinting compatibility. Experts added square bounding boxes around spheroids and annotated them as “high-quality”, if spheroids were suitable for the extrusion-based bioprinting process, or “low-quality” otherwise (Figure 3B). Figure 3B shows examples of “high-quality” and “low-quality” tri-cellular spheroids based on size, shape, viability, compactness, and aggregation. The tri-cellular dataset consisted of 516 images with 4,370 tri-cellular spheroids, 95% of which were manually annotated as “high-quality” by experts. The smaller size of this dataset was due to the limited availability of tri-culture spheroids used for bioprinting. To compensate for the small tri-culture training dataset, we created an HepG2 dataset consisting of 1,395 images featuring 19,129 HepG2 spheroids, 65.9% of which were annotated as “high-quality” and the remaining as “low-quality” (see *Material and methods* section).

#### 3.4.3 Classification results

To assess the performance of our newly developed model, we used a test set of 52 new images with 459 spheroids. The confusion matrix of pre-trained and non-pre-trained models for the same test set are illustrated in Figure 3C, where model predictions for each class are compared to the ground truth established by expert labeling. The background column represents spheroids labeled by the experts but not detected by the model. The confusion matrices reveal that both models were able to correctly identify high-quality spheroids with 95% of accuracy, while the accuracy of low-quality spheroids increased from 69% to 75% when using transfer learning in the training pipeline. Knowing the elevated class imbalance in our dataset, we further assessed the performance of our pre-trained model by looking at precision and recall metrics. While precision is used to assess how reliable predictions for the class of interest (positive class) are, recall characterizes the performance of a model to correctly retrieve all relevant elements (Refer to Supplementary Figure S2 for metrics formulas). Figure 3D shows the plot of precision and recall when each of the two classes, high-quality (green lines) and low-quality (purple lines) spheroids, is considered the class of interest (positive class). Continuous lines indicate the results for the model trained with transfer learning while dashed lines correspond to the results obtained when the YOLOv5 network is solely trained on the tri-cellular dataset. Optimal pairs of precision and recall for each model and class are indicated with a star-shaped marker. Both models featured precision and recall scores above 95% for high-quality spheroids (green lines). Our pre-trained model demonstrated a precision and recall of 71% and 75% respectively in identifying low-quality spheroids (purple solid line), in contrast to the non-pre-trained model, which yielded scores of 55% and 69% for the same respective metrics. When considering the SpheroidSorter workflow, it is important to prevent low-quality spheroids from being left in the culture plate, which implies reducing the number of false negatives when low-quality spheroids are considered as the positive class. Therefore, we successfully increased the recall score for low-quality spheroids for the pre-trained model as shown in Figure 3D. These results suggest the efficacy of transfer learning in improving classification performance, particularly for the less-represented class of low-quality spheroids.

To gain deeper insights into the disparity in classification accuracy between low-quality and high-quality spheroids, we assessed the inter-rater agreement score among four different labelers, using a subset of 611 tri-cellular spheroids. The resulting 63% agreement score highlights the complexity of characterizing spheroid quality for bioprinting solely based on brightfield images. We further analyzed misclassified spheroids by our pre-trained network. As illustrated by examples in Figure 3E, the model exhibited a tendency to misclassify low-quality spheroids, particularly in cases where compactness and neighboring cells posed challenges in distinguishing between the two classes.

Overall, we successfully trained a neural network to detect and classify tri-cellular spheroids for bioprinting, using transfer learning to enhance classification accuracy when the availability of the spheroid model of interest was limited.

### 3.5 Characterization of the platform using HepG2 spheroids

**FIGURE 4:**
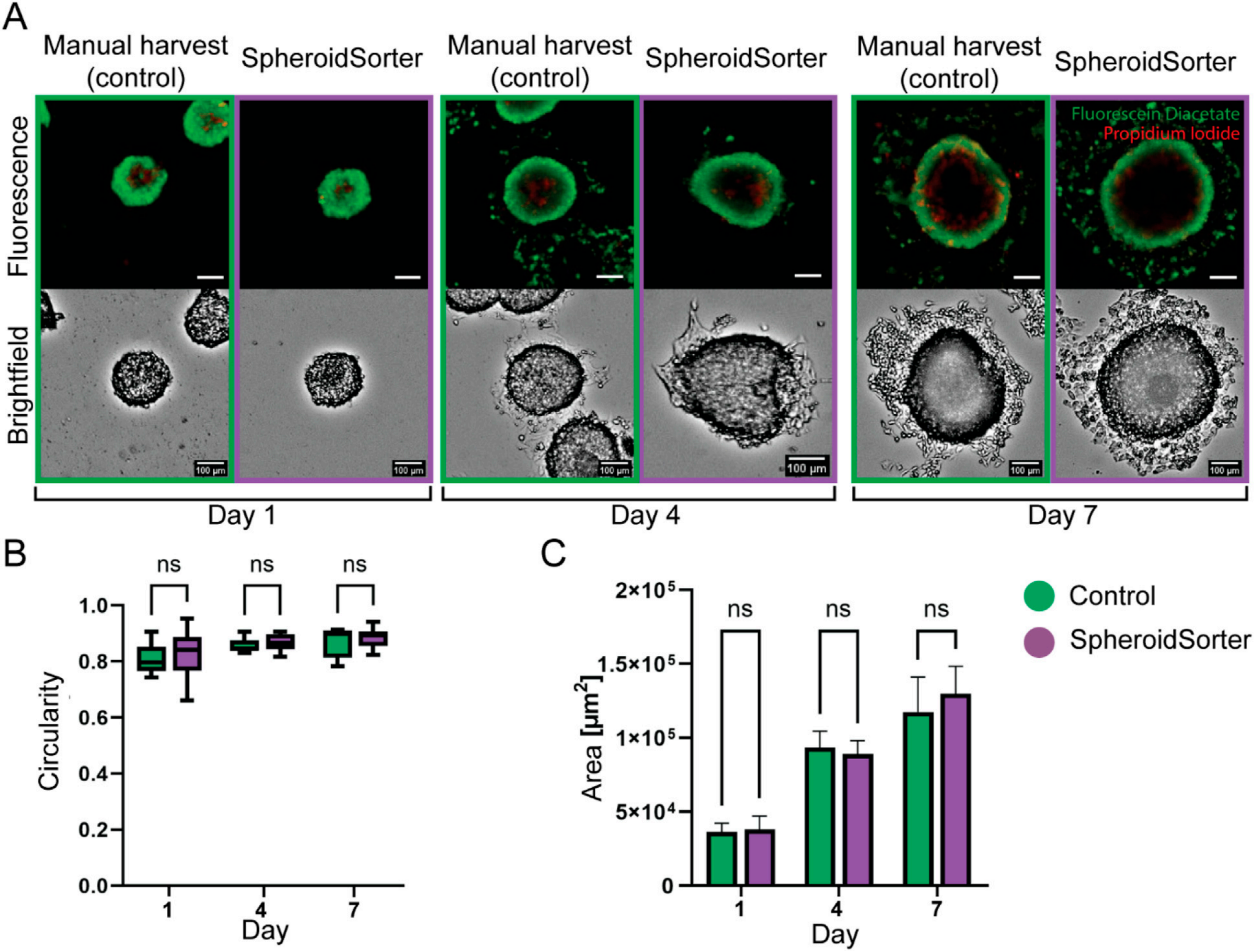
Viability assessment of HepG2 spheroids after manual harvest (Control) and automated harvest with the SpheroidSorter. **(A)** Fluorescent and bright-field images of HepG2 spheroids, showing spatial organization and proportion of dead cells (Propidium iodide, red) and live cells (Fluorescein Diacetate, green) in spheroids at day 1, 4, and 7 after harvest. Scale bar: 100 µm. **(B)** Area and **(C)** circularity of spheroids harvested with the SpheroidSorter platform were compared to those harvested manually (Control) at day 1, 4, and 7 after harvest. Mean ± SEM, n = 10–20 spheroids per condition.

The viability of spheroids after an automated sorting process is a crucial criterion of success. We used HepG2 spheroids to characterize our system and to obtain initial insights into the impact of using the SpheroidSorter platform with 3D tissues. HepG2 spheroids were subjected to a manual (control) as well as automated sorting process with the SpheroidSorter platform, and the sorting impact was assessed by morphological analysis and fluorescent live/dead staining on day 1, 4, and 7 after harvest. Fluorescent live/dead staining showed that the spatial localization and proportion of dead cells, stained with Propidium Iodide (PI, red), and live cells, stained with Fluorescein Diacetate (FDA, green), were comparable between manual and automated sorting (Figure 4A). For all three time points, dead cells were mostly observed at the center of the spheroids, indicating the development of a necrotic core, a well-known characteristic of aged spheroids (Štampar et al., 2020). Morphological analysis of brightfield images showed spherical and compact tissues for both conditions. We further characterized the impact of the SpheroidSorter platform by monitoring the circularity and area of harvested spheroids (Figure 4B). We observed similar circularity values for both manual and automated conditions at all three time points. In parallel, the mean spheroid area increased at a similar rate for both conditions, starting with 36.4 ± 2.8 E + 3 μm^2^ (control) and 38 ± 4.4 E + 3 μm^2^ (SpheroidSorter) on day 1 and reaching 118 ± 11.2 E + 3 μm^2^ (control) and 130 ± 8.9 E + 3 μm^2^ (SpheroidSorter) on day 7. These results suggest that spheroids retained viability and proliferation capacity after automated harvesting and, thus, that our harvesting process did not impact viability of HepG2 spheroids over 7 days compared to standard manual harvest.

### 3.6 Viability assessment of tri-cellular spheroids for bioprinting applications

To assess the use of the SpheroidSorter platform for bioprinting and, specifically, in the context of the OrganTrans project, we evaluated the impact of our platform on tri-cellular spheroids. These spheroids consisted of hepatic cells, mesenchymal cells and human umbilical cord endothelial cells (HUVECs), to increase hepatocyte functionality in *in vitro* models (Harrison et al., 2021; Jin et al., 2022). Within the OrganTrans bioprinting process, tri-cellular spheroids were cultured during 5 days in SP5D 24-well plates, before being sorted and mixed with a hydrogel, creating a bio-ink ready for immediate bioprinting. After printing, the liver construct was kept in a maturation reactor for 24 h.

**FIGURE 5:**
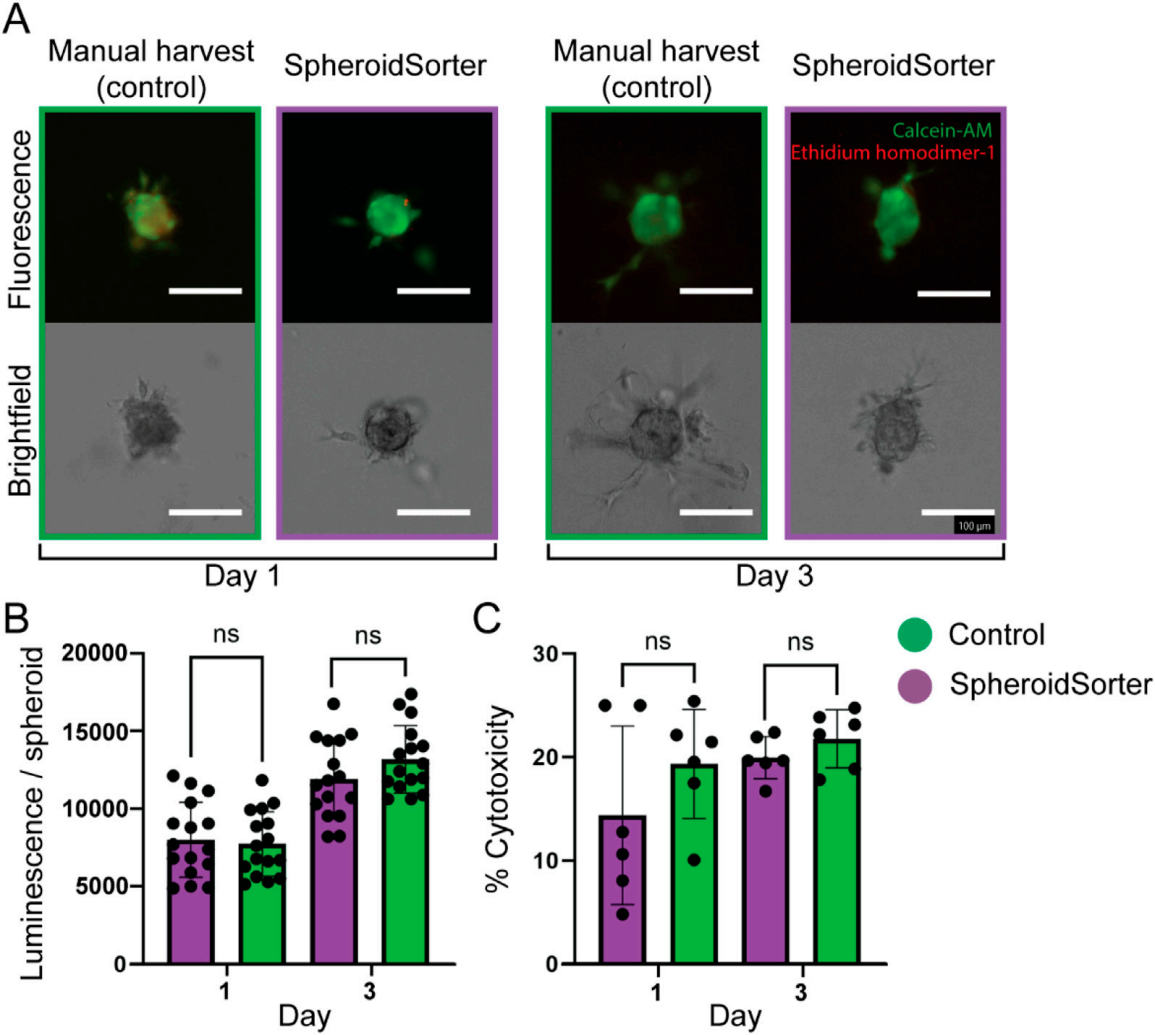
Viability assessment of tri-cellular spheroids after manual harvest (Control) and automated harvest with the SpheroidSorter. **(A)** Fluorescent and bright-field images of tri-cellular spheroids, showing spatial organization and proportion of dead cells (Ethidium homodimer-1, red) and live cells (Calcein-AM, green) in spheroids at day 1 and 3 after harvest. Scale bar: 100 µm. **(B)** ATP and **(C)** LDH measurements for tri-cellular spheroids harvested with the SpheroidSorter platform were compared to those harvested manually (Control) at day 1 and 3 after harvest for two independent experiments. Mean ± SEM, n = 50 spheroids per measure for ATP assay and n = 300 spheroids per measure for LDH assay.

We assessed the impact of the SpheroidSorter on tri-cellular spheroids by evaluating spheroid viability after manual and automated harvest over 3 days. To replicate the bio-ink physical characteristics more faithfully, we embedded spheroids in Matrigel for the fluorescent live/dead staining assay. We further assessed cell damage by measuring LDH release into the media and metabolic activity through intracellular ATP levels. Brightfield images of the spheroids embedded in Matrigel showed comparable growth and sprout formation for both the manual control and the SpheroidSorter condition, as shown in Figure 5A. Concurrently, fluorescent live/dead staining confirmed the absence of a necrotic core for both conditions after 3 days of culturing in hydrogel. Moreover, both conditions exhibited a similar increase in ATP levels per spheroid as shown in Figure 5B, starting with luminescence values at 9,476 ± 634 (control) and 9,836 ± 872 (SpheroidSorter) on day 1 and reaching 14,450 ± 1,073 (control) and 13,514 ± 1,046 (SpheroidSorter) on day 3. These results suggested that the SpheroidSorter platform did not impact spheroid growth after harvest. Additionally, Figure 5C shows that LDH levels were statistically similar between the two conditions at day 1 and 3, with percentages of cytotoxicity at 19.3% ± 2.2% and 21.8% ± 1.2% respectively for the control and at 14.4% ± 3.7% and 20.0% ± 0.9% for the SpheroidSorter condition, demonstrating that the automated handling of spheroids with our platform did not increase cytotoxicity.

In summary, these results suggest that the SpheroidSorter has no significant impact on spheroid viability and growth rates of tricellular liver spheroids 3 days after harvesting.

## 4 Discussion

Access to large quantities of homogeneous tissue models, such as spheroids, is essential for bioprinting complex organ structures intended for transplantation. However, persistent variability in large-scale production processes remains a primary challenge for effectively utilizing spheroids with bioprinting technology. In this study, we built a fully automated spheroid sorting platform that enabled high-throughput sorting of large quantities of spheroids while relying on deep learning-based image analysis to evaluate spheroid compatibility with bioprinting. This all-in-one system included imaging, analysis, and sorting of spheroids, thereby enhancing homogeneity within the spheroid population, which is critical for the viability of bioprinted organs.

Commercially available systems tackle the need for automated analysis and sorting of spheroids by scaling up standard single-cell isolation methods. These approaches rely on a pick-and-place strategy, analyzing and picking spheroids one by one. While these systems enable individual spheroid handling from standard culture plates, they fall short in providing compatibility with high-throughput processes due their slow handling procedure. In our SpheroidSorter platform, we have strategically limited the pick-and-place operation to low-quality spheroids, thereby minimizing platform movements. By using an efficient three-step workflow, we simultaneously imaged and analyzed all spheroids in the culture plate, allowing us to optimize the path for spheroid picking, which substantially increased the sorting speed. As a result, our average picking time was 5 s per spheroid, and the harvesting system could collect up to 8,000 high-quality spheroids in 20 min.

Alternative approaches for achieving high-throughput spheroid sorting involve fluorescence-activated cell sorting (FACS)-based systems, which were reported to achieve rates up to 50 spheroids per second (SpheroOne, Cellenion; COPAS FP, Union Biometrica). However, in contrast to the SpheroidSorter, the employed analysis method of the FACS systems is not compatible with bioprinting requirements because those systems rely on invasive fluorescent labels. To our knowledge, the COPAS VISION 250 (Copas Vision, Union Biometrica) is the only system that features brightfield imaging in a flow cytometry-based spheroid sorter, however it still relies on simple metrics, such as spheroid size and shape as sorting criteria. Our three-step workflow circumvents the need for fast analysis methods, such as laser-based FACS systems, and seamlessly integrates high-throughput sorting with label-free analysis of multiple parameters from brightfield images.

Another prerequisite for bioprinting applications is the use of distinct morphological parameters and viability levels as sorting parameters. This prerequisite poses a challenge for label-free analysis methods, as the gold standard for viability assessment relies on fluorescence. In recent years, machine learning has proven to be a powerful tool for spheroid analysis, particularly when used on brightfield images. So far, deep learning-based methods in sorting platforms have been mostly used for improving spheroid segmentation in brightfield images before manual feature selection (Grexa et al., 2021). While the segmentation of spheroids has proven to be a valuable tool to monitor spheroid growth and behavior (Chen et al., 2021), this approach faces challenges by blurred spheroid boundaries and when spheroid surroundings, such as floating single cells, influence spheroid classification. However, studies have demonstrated that allowing deep learning algorithms to identify crucial features in brightfield images can overcome manual selection of morphological parameters, enabling the classification of spheroids based on their viability (Zhang et al., 2019) and internal structures (Abdul et al., 2020; Trossbach et al., 2023), while circumventing missegmentation issues and segmentation biases. While promising, this method requires large training datasets of at least 15,000 images, limiting suitability for tissue models with limited available data. In our platform, we effectively employed deep learning to directly classify spheroids for bioprinting based on complex criteria and demonstrated that we can fine-tune our model using less than 5,000 tri-cellular liver spheroids by using transfer learning. Nonetheless, additional research studies should investigate the transferability potential of our model to other spheroid types and evaluate the necessity of biological models having morphological similarity.

While our model yielded remarkable results in sorting tri-cellular liver spheroids with a limited training dataset, there remains room for improvement in classification accuracy. A weakness of our training pipeline is the labeling strategy carried out by experts, assessing bioprinting compatibility based on brightfield images, despite the gold standard for viability characterization being fluorescence imaging. Despite the expert experience, this task remains challenging as indicated by the low inter-rater agreement score. To overcome this limitation, studies (Benning et al., 2020; Srisongkam et al., 2023) have integrated fluorescence-based viability measurements into the labeling pipeline. The fluorescence measurements provided a foundation for labeling brightfield images, enhancing the robustness of image classification. The trained model was then used to classify label-free spheroids based solely on brightfield images. This approach holds promise for enhancing our system, featuring high precision and accuracy in spheroid viability classification. Additionally, accuracy could be improved by using specific classification models, which are better adapted to our use case. Several studies have shown how improvements of the YOLOv5 structure and specific data augmentation methods have enhanced detection performances for small objects and have improved accuracy for unbalanced datasets (Wang et al., 2022; Liu et al., 2022). Moreover, among the several new versions of the YOLO series, YOLOv7 seems to surpass previous object detection models in speed and accuracy while also displaying faster training on small datasets without the need for pre-trained weights (Wang et al., 2022). According to other studies, spheroid classification could also benefit from combining multiple CNN-models with different performances and weighting the final decision with multiple individual network results (Park et al., 2023). Alternatively, using unsupervised learning for spheroid classification could provide different insights into spheroid characteristics by unveiling new patterns while reducing time-consuming labeling tasks.

The transferability potential of our sorting system to other spheroid types is mainly limited by the classification network. In this study we showed that transfer learning facilitates the classification of tri-culture liver spheroids using HepG2 spheroids that present similar morphology in the same culture plate. However, a more substantial dataset would be required to adapt the classification model for the analysis of spheroids produced in different culture plates. Another sensitive point for the transferability of our system is the shear stress that spheroids are exposed to during the harvesting process, especially in the custom-Falcon tube lid. Studies should first assess the impact of the SpheroidSorter platform on new spheroid types, similarly to what we conducted on tri-cellular spheroids. Overall, the design of our automated system makes it versatile to be used with different types of spheroids and culture plates. Fluidic tubing can be easily changed to match bigger spheroid size, and the plate holder is compatible with different standard plates. Moreover, despite the movement of the plate during sorting, the compatibility of our system with real-time imaging could compensate for spheroid displacement and thus make sorting from plates where tissue position is not fixed possible.

In summary, our developed platform offers an innovative solution for efficiently sorting large quantities of spheroids. Our meticulously designed three-step workflow, with an optimized use of a pick-and-place strategy, significantly reduced sorting time, which facilitates high-throughput sorting while maintaining high-accuracy picking. By employing deep learning-based analysis, we introduce a label-free method to evaluate spheroid compatibility for bioprinting, including viability assessment. Additionally, our findings demonstrate that transfer learning enabled deep learning-based image analysis even with small datasets of spheroids by using knowledge from models previously trained on similar tasks. This integration of deep learning analysis into high-throughput sorting processes holds great potential for significantly improving the sorting of tissue models. We foresee that the SpheroidSorter platform will give access to large populations of homogeneous spheroids, suitable for a variety of tissue engineering applications.

## Data Availability

The datasets generated for this study can be found in online Zenodo repositories with the following doi: 10.5281/zenodo.11121371, 10.5281/zenodo.11126980.

## References

[B2] Abdul L. Rajasekar S. Lin D. S. Y. Venkatasubramania Raja S. Sotra A. Feng Y. (2020). Deep-LUMEN assay - human lung epithelial spheroid classification from brightfield images using deep learning. Lab. Chip 20 (24), 4623–4631. 10.1039/d0lc01010c 33151236

[B3] Banerjee D. Singh Y. P. Datta P. Ozbolat V. O'Donnell A. Yeo M. (2022). Strategies for 3D bioprinting of spheroids: a comprehensive review. Biomaterials 291, 121881. 10.1016/j.biomaterials.2022.121881 36335718

[B4] Benning L. Peintner A. Finkenzeller G. Peintner L. (2020). Automated spheroid generation, drug application and efficacy screening using a deep learning classification: a feasibility study. Sci. Rep. 10, 11071. 10.1038/s41598-020-67960-0 32632214 PMC7338379

[B5] Bian X. Li G. Wang C. Liu W. Lin X. Chen Z. (2021). A deep learning model for detection and tracking in high-throughput images of organoid. Comput. Biol. Med. 134, 104490. 10.1016/j.compbiomed.2021.104490 34102401

[B6] Brandenberg N. Hoehnel S. Kuttler F. Homicsko K. Ceroni C. Ringel T. (2020). High-throughput automated organoid culture via stem-cell aggregation in microcavity arrays. Nat. Biomed. Eng. 4, 863–874. 10.1038/s41551-020-0565-2 32514094

[B7] Chen Z. Ma N. Sun X. Li Q. Zeng Y. Chen F. (2021). Automated evaluation of tumor spheroid behavior in 3D culture using deep learning-based recognition. Biomaterials 272, 120770. 10.1016/j.biomaterials.2021.120770 33798957

[B8] Ching T. Himmelstein D. S. Beaulieu-Jones B. K. Kalinin A. A. Do B. T. Way G. P. (2018). Opportunities and obstacles for deep learning in biology and medicine. J. R. Soc. Interface 15, 20170387. 10.1098/rsif.2017.0387 29618526 PMC5938574

[B9] CSEM (2024). Visard. Available at: https://www.csem.ch/en/news/csem-visard/(Accessed April 09, 2024).

[B10] Du X. Chen Z. Li Q. Yang S. Jiang L. Yang Y. (2023). Organoids revealed: morphological analysis of the profound next generation *in-vitro* model with artificial intelligence. Biodes. Manuf. 6 (3), 319–339. 10.1007/s42242-022-00226-y 36713614 PMC9867835

[B11] GBD 2017 Cirrhosis Collaborators Safiri S. Bisignano C. Ikuta K. S. Merat S. Saberifiroozi M. (2020). The global, regional, and national burden of cirrhosis by cause in 195 countries and territories, 1990-2017: a systematic analysis for the Global Burden of Disease Study 2017. Lancet Gastroenterol. Hepatol. 5 (3), 245–266. 10.1016/S2468-1253(19)30349-8 31981519 PMC7026710

[B12] Grexa I. Diosdi A. Harmati M. Kriston A. Moshkov N. Buzas K. (2021). SpheroidPicker for automated 3D cell culture manipulation using deep learning. Sci. Rep. 11, 14813. 10.1038/s41598-021-94217-1 34285291 PMC8292460

[B13] Gritti N. Lim J. L. Anlaş K. Pandya M. Aalderink G. Martínez-Ara G. (2021). MOrgAna: accessible quantitative analysis of organoids with machine learning. Development 148, dev199611. 10.1242/dev.199611 34494114 PMC8451065

[B14] Harrison S. P. Baumgarten S. F. Verma R. Lunov O. Dejneka A. Sullivan G. J. (2021). Liver organoids: recent developments, limitations and potential. Front. Med. 8, 574047. 10.3389/fmed.2021.574047 PMC8131532 34026769

[B15] Jin B. Liu Y. Du S. Sang X. Yang H. Mao Y. (2022). Current trends and research topics regarding liver 3D bioprinting: a bibliometric analysis research. Front. Cell Dev. Biol. 10, 1047524. 10.3389/fcell.2022.1047524 36518541 PMC9742412

[B16] Jocher G. Chaurasia A. Stoken A. Borovec J. Kwon Y. Michael K. (2022). ultralytics/yolov5: v7.0 - YOLOv5 SOTA Realtime Instance Segmentation. Zenodo. 10.5281/zenodo.7347926

[B17] Kalbhor M. Shinde S. Wajire P. Jude H. (2023). CerviCell-detector: an object detection approach for identifying the cancerous cells in pap smear images of cervical cancer. Heliyon 9, e22324. 10.1016/j.heliyon.2023.e22324 38058644 PMC10696000

[B18] Kupiec-Weglinski J. W. (2022). Grand challenges in organ transplantation. Front. Transpl. 1 1, 897679. 10.3389/frtra.2022.897679 PMC11235338 38994397

[B19] Lacalle D. Castro-Abril H. A. Randelovic T. Domínguez C. Heras J. Mata E. (2021). SpheroidJ: an open-source set of tools for spheroid segmentation. Comput. Methods Programs Biomed. 200, 105837. 10.1016/j.cmpb.2020.105837 33221056

[B20] Liu H. Sun F. Gu J. Deng L. (2022). SF-YOLOv5: a lightweight small object detection algorithm based on improved feature fusion mode. Sensors 22 (15), 5817. 10.3390/s22155817 35957375 PMC9371183

[B21] Liu S. Qi L. Qin H. Shi J. Jia J. (2018). Path aggregation network for instance segmentation. Available at: https://arxiv.org/abs/1803.01534 (Accessed August 13, 2024).

[B22] Liu Y. B. Chen M. K. (2022). Epidemiology of liver cirrhosis and associated complications: current knowledge and future directions. World J. Gastroenterol. 28 (41), 5910–5930. 10.3748/wjg.v28.i41.5910 36405106 PMC9669831

[B23] Matthews J. M. Schuster B. Kashaf S. S. Liu P. Ben-Yishay R. Ishay-Ronen D. (2021). OrganoID: a versatile deep learning platform for tracking and analysis of single-organoid dynamics. PLoS Comput. Biol. 18, e1010584. 10.1371/journal.pcbi.1010584 PMC9645660 36350878

[B24] Mironov V. Visconti R. P. Kasyanov V. Forgacs G. Drake C. J. Markwald R. R. (2009). Organ printing: tissue spheroids as building blocks. Biomaterials 30 (12), 2164–2174. 10.1016/j.biomaterials.2008.12.084 19176247 PMC3773699

[B25] Park S. Chien A. L. Lin B. Li K. (2023). FACES: a deep-learning-based parametric model to improve rosacea diagnoses. Appl. Sci. 13, 970. 10.3390/app13020970 38282829 PMC10817774

[B26] Petrosyan A. Montali F. Peloso A. Citro A. Byers L. N. La Pointe C. (2022). Regenerative medicine technologies applied to transplant medicine. An update. Front. Bioeng. Biotechnol. 10, 1015628. 10.3389/fbioe.2022.1015628 36263358 PMC9576214

[B27] Ramadan Q. Zourob M. (2021). 3D bioprinting at the frontier of regenerative medicine, pharmaceutical, and food Industries. Front. Med. Technol. 2, 607648. 10.3389/fmedt.2020.607648 35047890 PMC8757855

[B28] Redmon J. Divvala S. K. Girshick R. B. Farhadi A. (2015). “You only look once: unified, real-time object detection,” in 2016 IEEE conference on computer vision and pattern recognition (CVPR), 779–788. 10.1109/CVPR.2016.91

[B29] Redmon J. Farhadi A. (2018). YOLOv3: an incremental improvement. Available at: https://arxiv.org/abs/1804.02767 (Accessed August 13, 2024).

[B30] Renner H. Grabos M. Becker K. J. Kagermeier T. E. Wu J. Otto M. (2020). A fully automated high-throughput workflow for 3D-based chemical screening in human midbrain organoids. eLife 9, e52904. 10.7554/eLife.52904 33138918 PMC7609049

[B31] Renner H. Schöler H. R. Bruder J. M. (2021). Combining automated organoid workflows with artificial intelligence-based analyses: opportunities to build a new generation of interdisciplinary high-throughput screens for Parkinson's disease and beyond. Mov. Disord. 36 (12), 2745–2762. 10.1002/mds.28775 34498298

[B32] Schuster B. Junkin M. Kashaf S. S. Romero-Calvo I. Kirby K. Matthews J. (2020). Automated microfluidic platform for dynamic and combinatorial drug screening of tumor organoids. Nat. Commun. 11, 5271. 10.1038/s41467-020-19058-4 33077832 PMC7573629

[B33] Srisongkram T. Syahid N. F. Piyasawetkul T. Thirawatthanasak P. Khamtang P. Sawasnopparat N. (2023). Prediction of spheroid cell death using fluorescence staining and convolutional neural networks. Chem. Res. Toxicol. 36 (12), 1980–1989. 10.1021/acs.chemrestox.3c00257 38052002

[B40] Štampar M. Breznik B. Filipič M. Žegura B. (2020). Characterization of in vitro 3D cell model developed from human hepatocellular carcinoma (HepG2) cell line. Cells 9 (12), 2557. 10.3390/cells9122557 33260628 PMC7759933

[B34] Terrault N. A. Francoz C. Berenguer M. Charlton M. Heimbach J. (2023). Liver transplantation 2023: status report, current and future challenges. Clin. Gastroenterol. Hepatol. 21 (8), 2150–2166. 10.1016/j.cgh.2023.04.005 37084928

[B35] Trossbach M. Åkerlund E. Langer K. Seashore-Ludlow B. Joensson H. N. (2023). High-throughput cell spheroid production and assembly analysis by microfluidics and deep learning. SLAS Technol. 28 (6), 423–432. 10.1016/j.slast.2023.03.003 36990352

[B36] Ultralytics YOLO D. (2024). YOLOv5 architecture. Available at: https://docs.ultralytics.com/yolov5/tutorials/architecture_description/(Accessed August 13, 2024).

[B37] Wang A. Peng T. Cao H. Xu Y. Wei X. Cui B. (2022). TIA-YOLOv5: an improved YOLOv5 network for real-time detection of crop and weed in the field. Front. Plant Sci. 13, 1091655. 10.3389/fpls.2022.1091655 36618638 PMC9815699

[B38] Wang C. Y. Mark Liao H. Y. Yeh I. H. Wu Y. H. Chen P. Y. Hsieh J. W. (2019). CSPNet: a new backbone that can enhance learning capability of CNN. Available at: https://arxiv.org/abs/1911.11929 (Accessed August 13, 2024).

[B39] Zhang Z. Chen L. Wang Y. Zhang T. Chen Y. C. Yoon E. (2019). Label-free estimation of therapeutic efficacy on 3D cancer spheres using convolutional neural network image analysis. Anal. Chem. 91 (21), 14093–14100. 10.1021/acs.analchem.9b03896 31601098 PMC13134704

