# Supplementary material for: High-throughput platform for label-free sorting of 3D spheroids using deep learning
Source: Front Bioeng Biotechnol. 2024 Dec 9;12:1432737. doi: 10.3389/fbioe.2024.1432737 (PMC11663632; doi:10.3389/fbioe.2024.1432737)
Supplement: Supplementary file 1 [file DataSheet1.docx]

Supplementary Material

# Supplementary Figures


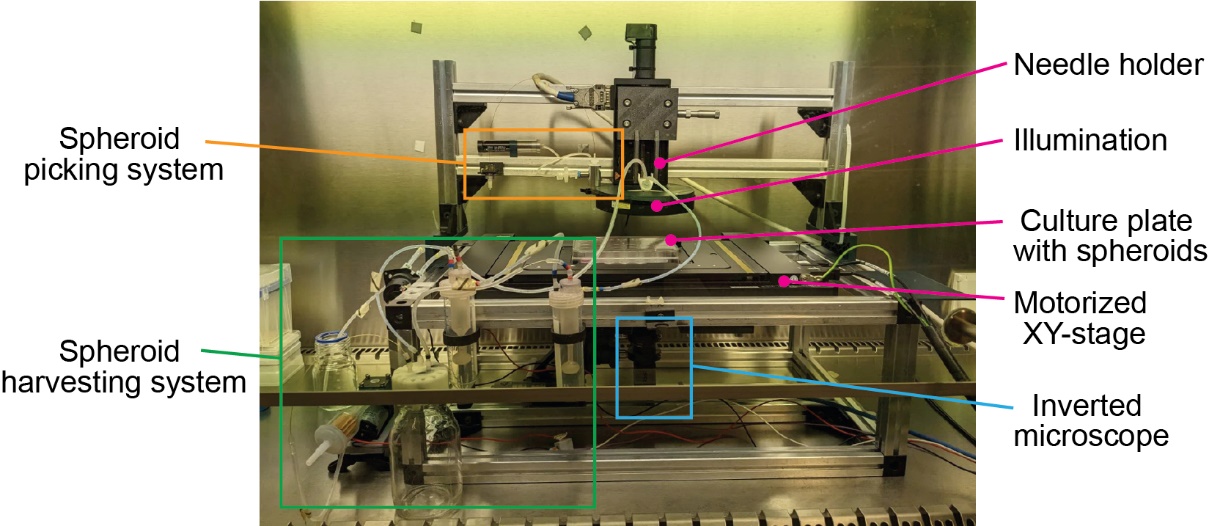


**Supplementary Figure 1.** SpheroidSorter platform. a) Photograph of the SpheroidSorter platform in a laminar flow cabinet. The platform is organized around a moving XY-stage, containing the SP5D culture plate with spheroids. It includes an inverted microscope, a fluidic system for spheroid picking and a fluidic system for spheroid harvesting.


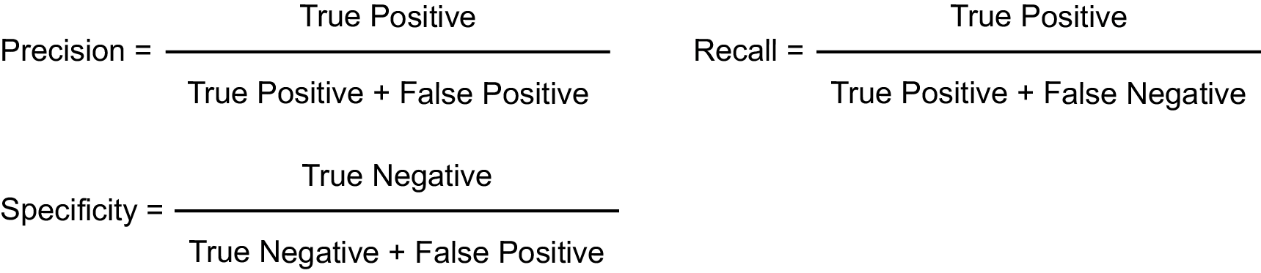


**Supplementary Figure 2.** Precision and recall formulas, where True Positive represents the number of correct predictions for the relevant class, False Positive is the number of incorrect predictions for the relevant class, True Negative is the number of correct predictions for the negative class and False Negative is the number of incorrect predictions for the negative class.
